# Axial Load Capacity Prediction of Concrete-Filled Steel Tubes Using Machine Learning: A Comparative Study

**DOI:** 10.3390/ma19112209

**Published:** 2026-05-24

**Authors:** Bingzhe Chen, Weining He, Lili Huang, Xianying Shi

**Affiliations:** College of Ocean and Civil Engineering, Dalian Ocean University, Dalian 116023, China; 15668932201@163.com (B.C.); h6_0213@163.com (W.H.)

**Keywords:** concrete-filled steel tubes, axial load capacity, eXtreme gradient boosting, graphical user interface

## Abstract

Concrete-filled steel tubes (CFSTs) combine the advantages of both steel and concrete and have been widely applied in recent years due to their excellent mechanical performance and economic efficiency. This paper focuses on the axial load capacity of CFST columns. First, 227 sets of experimental data were collected to develop axial load capacity prediction models using six machine learning algorithms. Second, the existing design code-recommended calculation models were evaluated. Subsequently, parameter importance and sensitivity analyses were conducted using the optimal machine learning model, and a graphical user interface was developed for predicting the axial load capacity of circular CFST columns. The results show that the eXtreme Gradient Boosting model is the most suitable for predicting the axial load capacity of CFST columns. In contrast, the stability and accuracy of the code-recommended models need improvement. The steel tube diameter and thickness have a significantly greater influence on axial load capacity compared to other parameters. Within specific ranges, increasing all parameters except column length enhances the axial load capacity.

## 1. Introduction

Concrete-filled steel tube (CFST) is a type of composite structural member consisting of a steel tube filled with concrete [1]. Due to its excellent mechanical properties and economic efficiency, CFST has been widely used in various civil engineering structures over the past few decades, particularly experiencing rapid development in high-rise buildings, long-span bridges, and underground engineering [2,3]. CFST structures combine the advantages of both steel and concrete, exhibiting high load-bearing capacity, good ductility, superior energy absorption capability, and enhanced fire resistance. Among these, circular CFST columns are the most widely applied, primarily because the surrounding steel tube effectively confines the core concrete in the transverse direction, significantly enhancing the strength and ductility of the concrete and improving its compressive behavior [4,5,6]. Compared with square or polygonal sections, circular CFST columns offer superior stiffness distribution and post-yield performance [7,8].

Furthermore, the construction process eliminates the need for additional formwork, simplifying construction procedures, shortening construction duration, and reducing building costs [9,10]. Extensive research indicates that the mechanical performance of CFST is significantly influenced by factors such as concrete strength, the diameter-to-thickness ratio of the steel tube, and the bond interaction at the steel–concrete interface. Therefore, CFST columns demonstrate promising application prospects and significant potential for widespread adoption in modern construction [11].

Research on the axial compression behavior of CFST columns has made significant progress over the past two decades, yet key mechanical mechanisms remain controversial. Existing studies indicate that the confinement effect of the steel tube on the core concrete is the core mechanism enhancing the load-bearing capacity of the member [12,13]. However, different scholars have reached divergent conclusions regarding the quantification methods of confinement strength and the influence of critical parameters such as member size effects, based on numerical simulations and theoretical analyses [14]. At the level of code application, although international mainstream design standards provide formulas for calculating the load capacity of CFST columns, their theoretical assumptions and applicable conditions show significant discrepancies [15,16]. Recent studies point out that these code-based models are primarily based on idealized material constitutive relationships, leading to prediction deviations from experimental data as high as 10–20% [17]. While experimental research serves as the foundation for theoretical validation, it faces challenges such as high cost, long duration, and technical barriers. Furthermore, measurement errors (e.g., strain gauge slippage) and specimen variability further affect data reliability. Although numerical simulations can reduce costs, they are susceptible to the choice of constitutive models.

Given these issues, computer-aided and computational-intelligence modeling for predicting the load-carrying capacity of concrete-filled steel tube columns has attracted increasing research attention, as demonstrated by recent studies on AI-powered GUI-based axial compression capacity prediction and computational-intelligence-based estimation for elliptical CFST columns [18,19]. In a broader structural engineering context, Spyridis and Olalusi (2021) developed machine-learning-based predictive models for the tensile breakout capacity of fastening systems in concrete using Gaussian process regression and support vector regression, further demonstrating the applicability of data-driven methods to concrete-related load-capacity assessment [20]. Nguyen et al. (2020) [21] collected experimental data from 99 rectangular CFST columns. They developed an axial load capacity prediction model using a feedforward neural network combined with an invasive weed optimization algorithm. Lyu et al. (2021) [22] gathered 478 sets of experimental data. They optimized a support vector machine model using a sine-cosine optimization algorithm to build a load capacity prediction model for circular CFST columns. Javed et al. (2020) [17] collected 227 sets of experimental data on circular CFST columns. They used gene expression programming to develop an explicit computational model for predicting the axial load capacity of CFST columns. These models have greatly advanced the research on the axial load capacity of CFST columns.

However, current models lack comparative studies of different models under the same database. They are mostly black-box models that do not effectively reveal the influence of various parameters on axial load capacity. Moreover, these models remain at the algorithmic level and are less convenient for engineers to use directly compared to formulas recommended by design codes. Based on these considerations, this study proposes to establish a database, employ six machine learning algorithms to develop prediction models for the axial load capacity of circular concrete-filled steel tubes, and create a user-friendly graphical user interface (GUI) for practical engineering applications.

Section 2 describes the data construction and statistical characteristics of the collected database. Section 3 presents the development and evaluation of the machine learning models and code-recommended models, as well as the parameter importance and sensitivity analyses. Section 4 introduces the developed graphical user interface. Section 5 summarizes the main conclusions.

## 2. Materials and Methods

**Figure 1:**
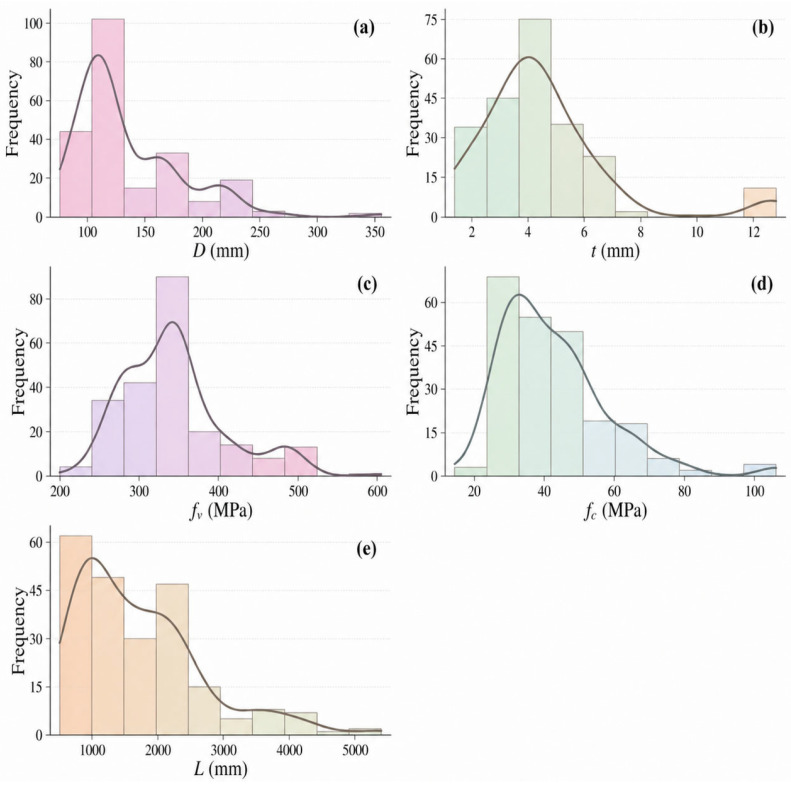
Parameter distribution.(**a**) steel tube diameter, D (mm); (**b**) steel tube wall thickness, t (mm); (**c**) steel yield strength, fy (MPa); (**d**) concrete compressive strength, fc (MPa); (**e**) column length, L (mm).

This study is based on the experimental data of axial load capacity of 227 CFST columns collected by Javed et al. (2020) [17]. In their research, six parameters were considered: steel tube diameter (D), steel tube wall thickness (t), steel yield strength ($f_{y}$), concrete compressive strength ($f_{c}$), column length (L), and the ratio of column length to steel tube diameter (L/D). The work of Javed et al. has made a positive contribution to the development of axial load capacity models for CFST columns. However, the sixth parameter, L/D, is a combination of D and L, which is unfavorable for constructing machine learning models. Therefore, this study considers only the first five parameters for subsequent machine learning model development. The distribution of each parameter is shown in Figure 1.

As shown in Figure 1, the steel tube diameter is primarily concentrated around 100 mm (with over 80 specimens), and very few specimens exceed 250 mm. The tube thickness is mainly distributed between 3 mm and 6 mm, with the most significant proportion falling between 4 mm and 5 mm. The steel yield strength is primarily in the range of 250 MPa to 500 MPa, with the majority of samples (over 80) falling between 300 MPa and 350 MPa. The concrete strength is mainly distributed between 20 MPa and 70 MPa, with the highest number of samples (over 60) in the range of 30 MPa to 40 MPa. The column length is uniformly distributed between 0 mm and 2500 mm, with a relatively low proportion of specimens exceeding 3000 mm.

## 3. Results

### 3.1. Machine Learning Algorithms

#### 3.1.1. Linear Regression

Linear Regression (LR) is a fundamental and widely used technique in supervised learning for modeling the relationship between a dependent variable (target) and one or more independent variables (features). It operates under the assumption that this relationship can be effectively approximated by a linear combination of the input features [23]. The goal is to find the optimal set of coefficients that minimizes the difference between predicted and actual outcomes, typically measured by the Mean Squared Error (MSE). This optimization can be achieved through analytical methods like the least squares approach or iterative algorithms such as gradient descent. Valued for its simplicity, interpretability, and computational efficiency, linear regression serves as a cornerstone in predictive modeling and data analysis.

#### 3.1.2. Support Vector Machine

Support Vector Regression (SVR) extends Support Vector Machine principles to regression, predicting continuous outcomes [24]. Unlike traditional methods that minimize all prediction errors, SVR uses an ε-insensitive loss function, which only penalizes errors larger than a threshold ε. Predictions within this ε-tube are treated as accurate, enhancing noise tolerance. This approach emphasizes generalization by balancing model complexity and error tolerance. SVR achieves robust performance by focusing on support vectors—samples outside the ε-margin. To handle nonlinear relationships, it employs kernel functions (e.g., RBF, polynomial) to map inputs into higher-dimensional spaces where a linear regression becomes feasible [24]. This kernel trick enables SVR to capture complex data patterns effectively. Due to its insensitivity to outliers and small fluctuations, SVR performs well in noisy environments or with non-normal error distributions. It is widely used in applications requiring high generalization and stability, such as financial forecasting and engineering modeling.

#### 3.1.3. Back-Propagation Neural Network

A backpropagation (BP) neural network is a fundamental deep learning model capable of learning complex, nonlinear input–output relationships [25]. It consists of an input layer, one or more hidden layers, and an output layer, with neurons in each layer connected by adjustable weights. During training, inputs are passed forward through the network to generate predictions. The resulting error is then propagated backward to compute gradients of the loss function with respect to each weight, using the chain rule of calculus. These gradients guide weight updates via optimization methods like gradient descent, gradually minimizing prediction error [26]. Nonlinear activation functions (e.g., ReLU, Sigmoid) enable the network to model intricate patterns through hierarchical feature representation. While BP networks excel in tasks like classification and regression, they require careful tuning of hyperparameters and are prone to overfitting. Nevertheless, their strong learning capability makes them widely used in diverse AI applications.

#### 3.1.4. Decision Tree

Decision Tree (DT) is an interpretable supervised learning algorithm used for classification and regression [27]. It recursively partitions the data by selecting the most informative features and optimal split points, creating a tree-like structure. Internal nodes represent feature tests, branches denote outcomes, and leaf nodes hold final predictions—class labels or continuous values. Splits are determined using criteria like information gain, Gini impurity, or variance reduction to maximize subset homogeneity. Key advantages include high interpretability, ease of visualization, and the ability to handle both numerical and categorical data with minimal preprocessing. Decision trees naturally capture nonlinear relationships and feature interactions. However, they are prone to overfitting, especially with deep trees, leading to poor generalization [28]. This limitation is often addressed through pruning or by integrating them into ensemble methods like Random Forests and Gradient Boosting. Despite their simplicity, decision trees serve as foundational components in many advanced machine learning models.

#### 3.1.5. Random Forest

Random Forest (RF) is a robust ensemble method that improves prediction accuracy and stability by combining multiple decision trees [28]. It uses bootstrap sampling to train each tree on different subsets of the data. In contrast, at each split, only a random subset of features is considered—introducing diversity and reducing overfitting. This dual randomness (in data and features) decreases tree correlation and enhances model generalization. For regression tasks, predictions are averaged across all trees, smoothing individual errors and improving reliability. RF handles high-dimensional data, manages missing values, and provides insights into feature importance [29]. It is effective for both classification and regression, and its inherent resistance to overfitting and insensitivity to outliers make it widely applicable in real-world scenarios. Due to its simplicity, versatility, and firm performance, Random Forest is a popular choice in machine learning for diverse applications ranging from finance to bioinformatics.

#### 3.1.6. eXtreme Gradient Boosting

XGBoost (eXtreme Gradient Boosting) is a highly efficient and scalable gradient boosting framework based on decision trees [4]. It builds models sequentially, with each new tree correcting the errors of the previous ones, forming a strong ensemble. Its key advantage lies in a regularized objective function that includes L1 and L2 penalties, effectively controlling model complexity and reducing overfitting. Unlike basic gradient boosting, XGBoost uses second-order gradients (Hessian) for more precise optimization, enabling faster convergence and higher accuracy. It also supports parallel processing, handles missing data efficiently, and optimizes memory usage. These enhancements make XGBoost fast, robust, and well-suited for large-scale and complex datasets. Widely adopted in data science competitions and real-world applications, it excels in tasks such as classification, regression, and ranking, offering superior predictive performance and generalization ability.

### 3.2. Model Development

Based on the 227 sets of experimental data from Section 2, six machine learning algorithms—LR, SVM, BP neural network, DT, RF, and XGBoost—were employed to develop prediction models for the axial load capacity of CFST columns. The hyperparameters for each model were determined using grid search combined with five-fold cross-validation, as shown in Table 1.

**Figure 2:**
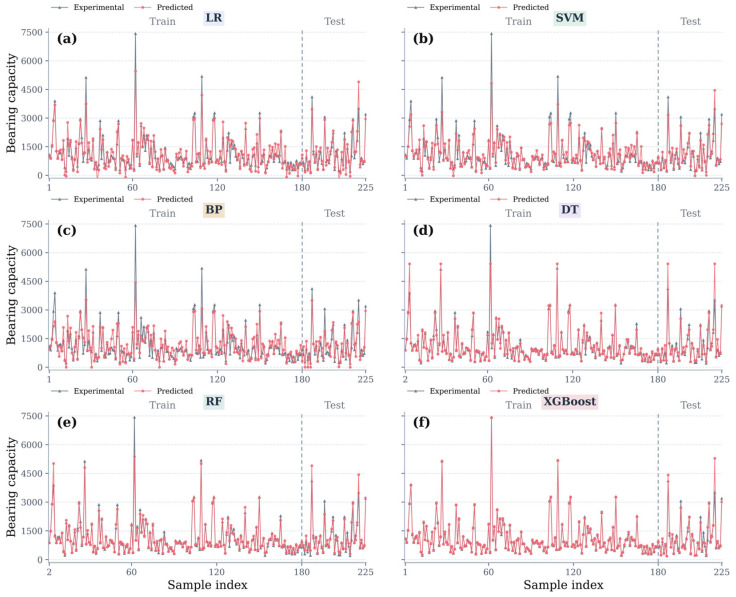
Experimental and predicted axial load capacities of CFST columns: (**a**) LR; (**b**) SVM; (**c**) BP; (**d**) DT; (**e**) RF; (**f**) XGBoost.

The differences between the predicted values and experimental values of each machine learning model on both the training set and the test set are shown in Figure 2.

As can be seen from Figure 2, compared to LR, SVM, and the BP neural network, the DT, RF, and XGBoost models exhibit minor discrepancies between predicted and experimental values. Moreover, among all models, XGBoost shows the best performance in predictions.

**Figure 3:**
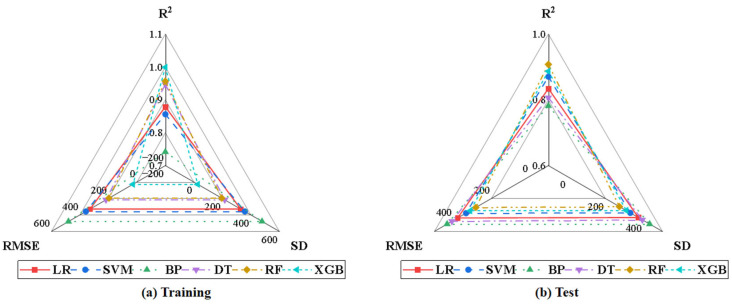
Model performance evaluation.

Given that the above observations might not be entirely clear, Figure 3 provides a detailed comparison of the different performances of various machine learning models across several performance metrics on both the training set and the test set. These performance metrics include R-squared (R2), standard deviation (SD), and root mean square error (RMSE). According to Figure 3, on the training set, XGBoost performs the best with the highest R2 and the lowest SD and RMSE. The performances of RF and DT are slightly lower than XGBoost, but still significantly better than the other models. The BP neural network model shows the worst performance in predicting the axial load capacity of CFST columns.

On the test set, RF exhibits the best performance with the highest R2 and the lowest SD and RMSE. XGBoost’s performance is slightly inferior to RF but still markedly better than the rest. Additionally, despite performing well on the training set, the DT model does not perform as well on the test set, only outperforming the BP neural network model. In conclusion, the XGBoost and RF models are most suitable for predicting the axial load capacity of CFST columns.

### 3.3. Evaluation of Researchers’ Models

In Section 3.1, machine learning models for predicting the axial load capacity of CFST columns were developed. To demonstrate their superiority, this section evaluates the existing design code-recommended formulas for calculating the axial load capacity of CFST columns. Models recommended by different codes are shown in Table 2. Due to the large number of parameters involved in the model and limitations on space, this study will not elaborate extensively. The code-recommended models were adopted from previous comparative studies and relevant design specifications [15,17,30,31,32].

**Figure 4:**
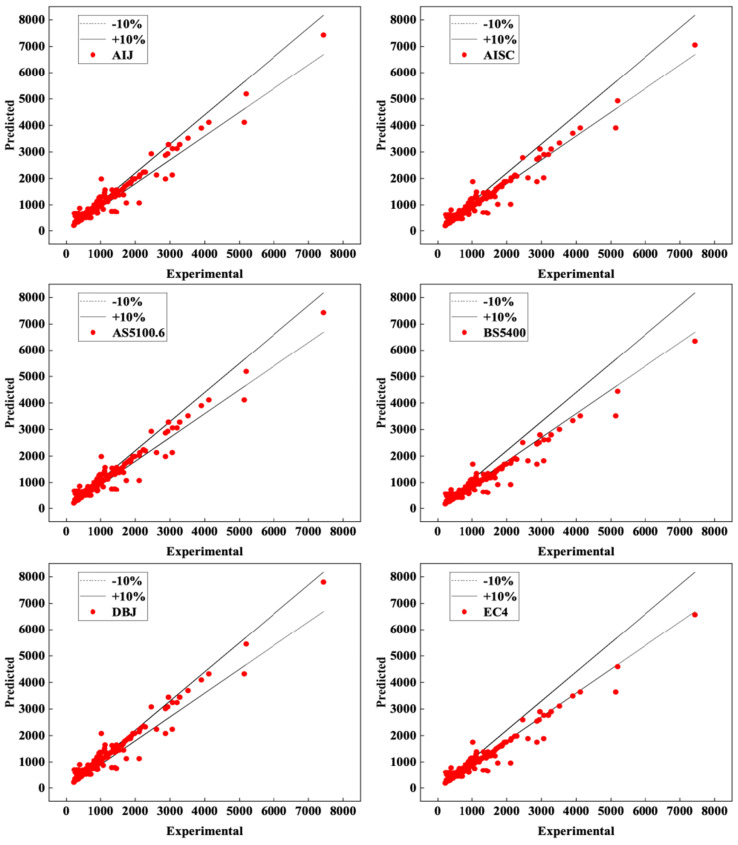
Calculated values of the code.

As shown in Figure 4, the code-recommended models show different degrees of deviation from the experimental values, indicating that their predictive performance varies across the collected database. Furthermore, Table 3 presents the minimum, maximum, SD, and coefficient of variation (COV) of the ratios between the calculated values from the code models and the experimental values. It can be observed from Table 3 that the ratios between predicted and experimental values vary significantly across different models, indicating a certain degree of instability in the code-recommended models. In addition, the COV between the calculated and experimental values for all code-recommended models exceeds 20%, revealing relatively large prediction errors.

**Figure 5:**
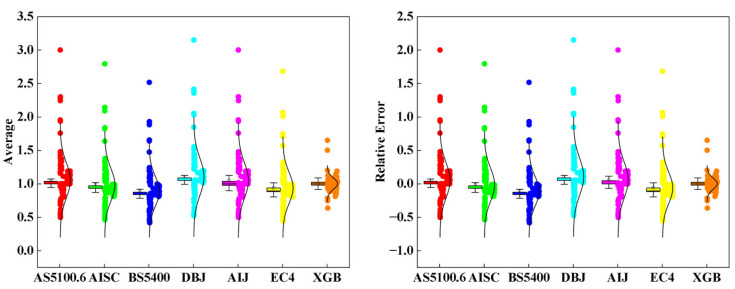
Performance of the code’s model.

Furthermore, Figure 5 presents the distribution of the mean values and relative errors between the calculated values from the code-recommended models and those from the XGBoost model developed in this study. The performance of the code-recommended models does not differ significantly among themselves, and the XGBoost model established in this study outperforms the existing recommended formulas. The mean ratio of the predicted values to experimental values for the XGBoost model is closer to 1, and its relative error is closer to 0.

### 3.4. Parameter Importance Analysis

**Figure 6:**
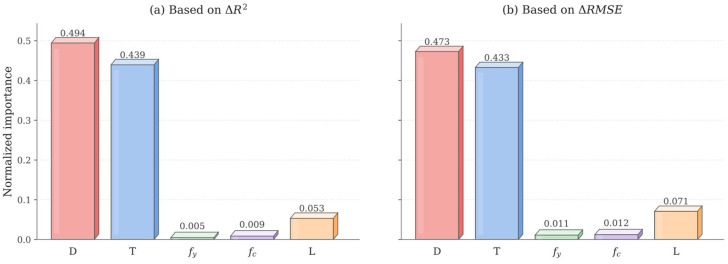
Parameter importance ranking.

As indicated in Section 3.1 and Section 3.2, the XGBoost model developed in this study demonstrates higher stability and accuracy compared to other machine learning models and the code-recommended models. This section employs the developed XGBoost model for parameter importance analysis to clarify the relative significance of different parameters on the axial load capacity of CFST columns. The parameter importance analysis is conducted as follows: when analyzing the importance of a specific parameter, that parameter is removed, and the model is retrained using the remaining parameters to make predictions, yielding a new R2 and RMSE. The importance of the parameter is then determined by the degree of degradation in the R-squared and RMSE. The rankings of parameter importance based on the R2 and RMSE are shown in Figure 6. From Figure 6, it can be concluded that the steel tube diameter (D) and steel tube thickness (t) are the most influential parameters on axial load capacity. The yield strength of the steel ($f_{y}$) and the compressive strength of the concrete ($f_{c}$) have relatively minor impacts, while the column length (L) has a greater influence than $f_{y}$ and $f_{c}$ but is significantly less critical than D and t.

### 3.5. Parameter Sensitivity Analysis

**Figure 7:**
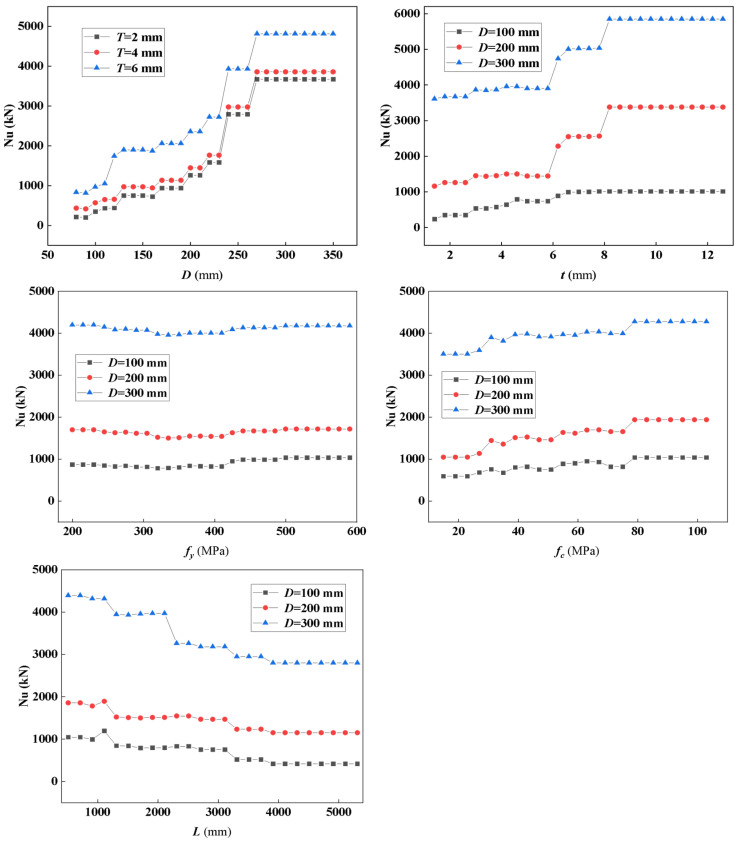
Parameter sensitivity.

Section 3.4 describes a parameter importance analysis. This section discusses a parameter sensitivity analysis using the developed XGBoost model. The sensitivity analysis procedure is as follows: when analyzing the sensitivity of a specific parameter, the values of all other parameters are set to their respective averages. The results of the sensitivity analysis for each parameter are shown in Figure 7.

From Figure 7, it can be observed that the steel tube diameter (D) has a positive effect on axial load capacity: generally, the larger the diameter, the higher the axial load capacity. However, when the diameter reaches a specific large value (around 300 mm), the axial load capacity of the CFST column tends to stabilize. This may be related to the range of D in the database constructed for this study and requires further investigation. The wall thickness (t) of the steel tube also has an overall positive effect on load capacity. However, when the tube diameter is small, increasing only the wall thickness results in a limited improvement in axial load capacity. Similar to the diameter, when the wall thickness reaches a specific large value (around 8 mm), further increases in thickness do not significantly enhance the axial load capacity.

The influence of steel yield strength ($f_{y}$) on axial load capacity shows a trend of first decreasing and then increasing, but the overall effect is not significant. The compressive strength of concrete ($f_{c}$) generally enhances the axial load capacity. However, when the concrete strength reaches a certain value (around 80 MPa), further increases in strength do not lead to significant gains in load capacity. Column length (L) is the only parameter that exhibits a negative effect on axial load capacity among all the parameters analyzed.

## 4. Graphical User Interface

**Figure 8:**
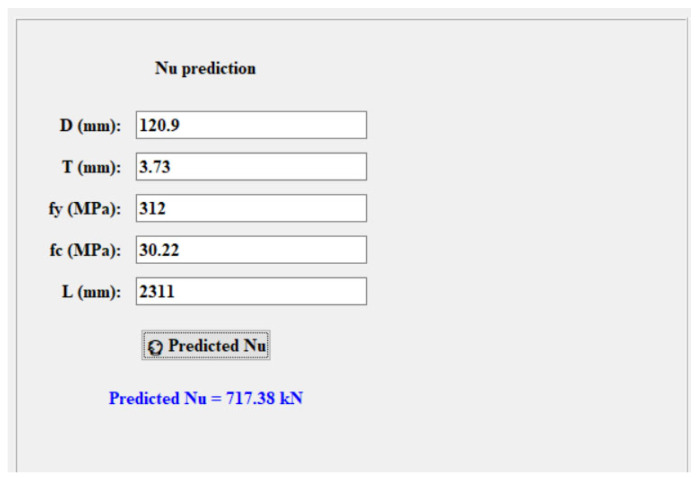
GUI for predicting the axial load-bearing capacity of CFST.

A graphical user interface (GUI) is a way that allows users to interact with electronic devices through graphical elements such as windows, icons, buttons, and other visual indicators. Compared to text-based user interfaces or command-line interfaces, a GUI provides a more intuitive and user-friendly method for operating software applications and operating systems. For non-technical users, directly interacting with command lines or code can be challenging. Through a GUI, users can interact with models via an intuitive interface without needing to understand the underlying technical details. Therefore, in this section, a GUI for predicting the axial load capacity of CFST columns is developed. As shown in Figure 8, simply inputting the required parameters allows for the calculation of the axial load capacity of a CFST column.

## 5. Conclusions

This study utilized existing experimental data and six machine learning algorithms to develop prediction models for the axial load capacity of CFST columns. The existing design code-recommended models were evaluated based on the experimental data. Parameter importance and sensitivity analyses were conducted using the optimal machine learning model developed in this study, leading to the following conclusions:

1. Among the machine learning models, both RF and XGBoost exhibited strong predictive performance. Although RF achieved the best performance on the test set, XGBoost showed more balanced performance across the training and test sets and was therefore selected as the optimal model for subsequent parameter importance analysis, sensitivity analysis, and GUI development.
2. The ratios between calculated values from the code-recommended models and experimental values exhibit large fluctuations, with coefficients of variation exceeding 20%, indicating that both stability and accuracy of these models need improvement. In terms of the distribution of mean values and relative errors, the XGBoost model developed in this study demonstrates superior stability and accuracy compared to the code-recommended models.
3. Steel tube diameter and wall thickness have a significantly greater influence on axial load capacity than the other parameters. Except for column length, which has a negative effect, all other parameters generally have a positive impact on axial load capacity. However, the enhancing effect of each parameter tends to plateau once it reaches a certain value.

All conclusions drawn in this study are based on a limited set of experimental data. The uneven distribution of parameters has been observed to significantly constrain the development and generalization capability of the machine learning models. Furthermore, the evaluation of the code-suggested models is solely based on predictive accuracy, failing to account for the inherent safety factors, calibration philosophy, and acceptable levels of conservatism embedded in design codes. Furthermore, the current approach primarily relies on data-driven patterns, lacking integration with the underlying mechanical principles of CFST columns. Future work should focus on collecting more comprehensive experimental data and incorporating domain-specific mechanistic knowledge to further optimize the models and enhance their physical interpretability and robustness.

## Figures and Tables

**Table 1:** Hyperparameters of the machine learning models.

Models	Hyperparameters
LR	-
SVM	kernel = ‘linear’, C = 10, epsilon = 0.01
BP	hidden_layer_sizes = 10, activation = ‘relu’, solver = ‘lbfgs’, max_iter = 200
DT	max_depth = 10, min_samples_split = 7, min_samples_leaf = 3
RF	n_estimators = 70, max_depth = 9, min_samples_split = 2, min_samples_leaf = 2
XGBoost	n_estimators = 100, learning_rate = 0.61, max_depth = 2

**Table 2:** Models recommended by codes.

Code	Ultimate Axial Moment Capacity
AIJ	$\begin{array}{ll} & N_{u1}=0.85A_{c}{f_{c}}^{\prime }+\left(1+\eta \right)A_{s}f_{y};\frac{l}{D}\leq 4\\ & N_{u2}=N_{u1}-0.125\left\{N_{u1}-N_{u3}\right\}\left(\frac{l}{D}-4\right);4<\frac{l}{D}\leq 12 \end{array}$ $\begin{array}{ll} & N_{u3}=A_{c}\sigma_{cr}+N_{crs};\frac{l}{D}>12\\ & \sigma_{cr}=\frac{1.7{f_{c}}^{\prime }}{1+\sqrt{\lambda_{1}^{4}+1}};\lambda_{1}\leq 1 \end{array}$ $\begin{array}{ll} & \sigma_{cr}=0.83exp\left\{\left(0.568+0.00612{f_{c}}^{\prime }\right)\left(1-\lambda_{1}\right)\right\}0.85{f_{c}}^{\prime };\lambda_{1}>1\\ & \lambda_{1}=\frac{\lambda }{\pi }\sqrt{0.93{\left(0.85{f_{c}}^{\prime }\right)}^{\frac{1}{4}}\times 10^{-3}} \end{array}$ $\begin{array}{ll} & N_{crs}=A_{s}f_{y};\lambda_{1}\leq 0.3\\ & N_{crs}=1-0.545\left(\lambda_{1}-0.3\right);0.3<\lambda_{1}\leq 1.3 \end{array}$ $\begin{array}{ll} & N_{crs}=\frac{N_{Es}}{1.3};\lambda_{1}>1.3\\ & \lambda_{1}=\frac{\lambda }{\pi }\sqrt{\frac{f_{y}}{Es}};N_{Es}=\frac{\pi^{2}E_{s}I_{s}}{l^{2}} \end{array}$
AS5100.6	$\begin{array}{ll} & N_{u}=\alpha_{c}\left[\eta_{a}A_{s}f_{y}+\left(1+\frac{\eta_{c}tf_{y}}{d_{0}{f_{c}}^{\prime }}\right)A_{c}{f_{c}}^{\prime }\right]\\ & \alpha_{c}=\xi \left(1-\sqrt{1-{\left(\frac{90}{\xi \lambda }\right)}^{2}}\right);\xi =\frac{{\left(\frac{\lambda }{90}\right)}^{2}+1+\eta }{2{\left(\frac{\lambda }{90}\right)}^{2}} \end{array}$ $\begin{array}{ll} & \lambda =\lambda_{n}+\alpha_{a}\alpha_{b};\eta =0.00326\left(\lambda_{n}-13.5\right)\geq 0;\lambda_{n}=90\lambda_{r}\\ & \lambda_{r}=\sqrt{\frac{N_{s}}{N_{cr}}};N_{s}=A_{s}f_{y}+A_{c}{f_{c}}^{\prime } \end{array}$ $\begin{array}{ll} & N_{cr}=\frac{\pi^{2}{\left(EI\right)}_{eff}}{l^{2}};{\left(EI\right)}_{eff}=E_{s}I_{s}+E_{c}I_{c}\\ & \alpha_{a}=\frac{2100\left(\lambda_{n}-13.5\right)}{\lambda_{n}^{2}-13.5\lambda_{n}+2050};\alpha_{b}=Presented\;in\;code\\ & \eta_{2}=0.25\left(3+2\lambda_{r}\right)\geq 0;\eta_{1}=4.9-18.5\lambda_{r}+17.5\lambda_{r}^{2}\geq 0 \end{array}$
AISC	$\begin{array}{ll} & N_{u}=\varphi_{c}N_{n};\varphi_{c}=0.75\left(\begin{array}{c} LRFD \end{array}\right)\\ & N_{n}=N_{o}\left[0.658^{\left(\frac{N_{o}}{N_{e}}\right)}\right];N_{e}\geq 0.44N_{o}\\ & N_{n}=0.877N_{e};N_{e}<0.44N_{o} \end{array}$ $\begin{array}{ll} & N_{o}=A_{s}f_{y}+0.95A_{c}{f_{c}}^{\prime }\\ & N_{e}=\frac{\pi^{2}{\left(EI\right)}_{eff1}}{{\left(KL\right)}^{2}};{\left(EI\right)}_{eff1}=E_{s}I_{s}+C_{1}E_{c}I_{c}\\ & C_{1}=0.1+2\left(\frac{A_{s}}{A_{c}+A_{s}}\right)\leq 0.3;E_{c}={\left({f_{c}}^{\prime }\right)}^{\frac{1}{2}} \end{array}$
BS5400	$\begin{array}{ll} & \alpha_{c}=\frac{0.45f_{cc}A_{c}}{N_{u}};0.1<\alpha_{c}<0.8\\ & N_{u}=0.91{f_{y}}^{\prime }A_{s}+0.45f_{cc}A_{c}\\ & {f_{y}}^{\prime }=C_{2}f_{y};f_{cc}=f_{cu}+C_{1}\frac{t}{D}f_{y} \end{array}$
DBJ	$\begin{array}{ll} & N_{u}=\gamma_{m}f_{scy}W_{scm}\\ & f_{scy}=\left(1.18+0.85\xi \right)f_{ck}\\ & W_{scm}=\frac{\pi }{32}D^{3}\\ & \xi =\frac{A_{s}f_{yk}}{A_{c}f_{ck}}\\ & \gamma_{m}=1.04+0.48ln\left(\xi +0.1\right) \end{array}$
EC4	$\begin{array}{ll} & N_{u}=\eta_{a}A_{s}f_{y}+\left(1+\eta_{c}\frac{t}{D}\frac{f_{y}}{{f_{c}}^{\prime }}\right)A_{c}{f_{c}}^{\prime }\\ & \eta_{a}=0.25\left(3+2\bar{\lambda }\right)\leq 1.0;\eta_{c}=4.9-18.5\bar{\lambda }+17{\bar{\lambda }}^{2}\geq 0\\ & \bar{\lambda }=\frac{N_{pIR}}{N_{cr}};N_{pIR}=A_{s}f_{y}+A_{c}{f_{c}}^{\prime }\\ & N_{cr}=\frac{\pi^{2}{\left(EI\right)}_{eff2}}{l_{2}};{\left(EI\right)}_{eff2}=E_{s}I_{s}+0.6E_{c}I_{c};\\ & E_{c}=22000{\left[\frac{\left({f_{c}}^{\prime }+8\right)}{10}\right]}^{0.3} \end{array}$

**Table 3:** Performance of the recommended models in the code.

Code	Min	Max	SD	CoV
AS5100.6	0.49	3	0.26	24.86%
AISC	0.46	2.79	0.24	24.76%
BS5400	0.41	2.51	0.22	24.77%
DBJ	0.52	3.14	0.27	24.84%
AIJ	0.49	3	0.26	25.11%

## Data Availability

The original contributions presented in this study are included in the article. Further inquiries can be directed to the corresponding authors.

## Abbreviations

The following abbreviations are used in this manuscript:

CFST	Concrete-Filled Steel Tubes
LR	Linear Regression
SVM	Support Vector Machine
SVR	Support Vector Regression
BP	Backpropagation Neural Network
DT	Decision Tree
RF	Random Forest
XGBoost	eXtreme Gradient Boosting
MSE	Mean Squared Error
RBF	Radial Basis Function
ReLU	Rectified Linear Unit
SD	Standard Deviation
RMSE	Root Mean Square Error
COV	Coefficient of Variation
GUI	Graphical User Interface
AIJ	Architectural Institute of Japan
AS5100.6	Australian Standard 5100.6
AISC	American Institute of Steel Construction
BS5400	British Standard 5400
EC4	Eurocode 4
